# Reverse swing‐M, phase 1 study of repurposing mebendazole in recurrent high‐grade glioma

**DOI:** 10.1002/cam4.3094

**Published:** 2020-05-13

**Authors:** Vijay M. Patil, Arti Bhelekar, Nandini Menon, Atanu Bhattacharjee, Vijai Simha, Ram Abhinav, Anuja Abhyankar, Epari Sridhar, Abhishek Mahajan, Ameya D. Puranik, Nilendu Purandare, Amit Janu, Ankita Ahuja, Rahul Krishnatry, Tejpal Gupta, Rakesh Jalali

**Affiliations:** ^1^ Department of Medical Oncology Tata Memorial Centre Homi Bhabha National Institute (HBNI) Mumbai India; ^2^ Section of Biostatistics Centre for Cancer Epidemiology Tata Memorial Centre Homi Bhabha National Institute (HBNI) Mumbai India; ^3^ Department of Pathology Tata Memorial Centre Homi Bhabha National Institute (HBNI) Mumbai India; ^4^ Department of Radiology Tata Memorial Centre Homi Bhabha National Institute (HBNI) Mumbai India; ^5^ Department of Nuclear Medicine Tata Memorial Centre Homi Bhabha National Institute (HBNI) Mumbai India; ^6^ Department of Radiation Oncology Tata Memorial Centre Homi Bhabha National Institute (HBNI) Mumbai India

**Keywords:** Checkpoint, Glioblastoma, High‐grade Glioma, Mebendazole, Recurrence, Repurposing, Salvage

## Abstract

**Background:**

Relapsed high‐grade glioma has dismal outcomes. Mebendazole has shown promising activity against glioma in in‐vitro and in‐vivo studies. Hence, we undertook a phase 1 study to repurpose mebendazole in the treatment of glioblastoma.

**Methods:**

We conducted a phase 1 study (accelerated titrated design 4) of mebendazole in patients with recurrent glioblastoma (GBM). Patients eligible for re‐irradiation were enrolled in arm A1 (radiation with concurrent temozolomide 75 mg/m^2^ daily during the course of radiation+mebendazole) while patients who were ineligible were enrolled in either arm B1 (CCNU 110 mg/m^2^ day 1, every 6 weekly + mebendazole) or arm C1 (temozolomide 200 mg/m^2^ day 1‐5, every 4 weekly + mebendazole). The primary endpoint of phase 1 was to identify the MTD of mebendazole in each combination.

**Findings:**

11 patients were enrolled in the whole study. MTD of mebendazole was not reached in arm A1 and C1 and hence the recommended dose for phase 2 was 1600 mg TDS (4800 mg) per day. The MTD of mebendazole in combination with CCNU was 1600 mg TDS (4800 mg) per day and the dose recommended for phase 2 was 800 mg TDS (2400 mg) per day. The three most common adverse events seen in the study were anemia (n = 9, 81.8%), nausea (n = 7, 63.6%), and fatigue (n = 6, 55.5%).

**Interpretation:**

The recommended phase 2 dose of mebendazole is 1600 mg TDS with temozolomide and temozolomide‐radiation combination while the dose of 800 mg TDS needs to be used with single‐agent CCNU.

## Graphical abstract

This was a phase 1 study in relapsed recurrent glioma for repurposing Mebendazole. The recommended dose identified for future studies for mebendazole in adult glioma patients was 4800 mg per day with both temozolomide‐radiation and single‐agent temozolomide while it was 2400 mg with CCNU.

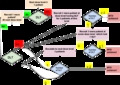

## 1 INTRODUCTION

Recurrence in glioblastoma (GBM) is inevitable. For patients with GBM treated with the current standard of care (maximal safe resection, fractionated external beam radiotherapy, and concurrent and adjuvant temozolomide) in the European Organisation for Research and Treatment of Cancer (EORTC)–National Cancer Institute (NCI) of Canada randomized trial, 2‐ and 5‐year progression‐free survival (PFS) of only 11% and 4%, respectively, were observed with less than 10% of patients surviving more than 5 years from diagnosis.^1^, ^2^ Management of recurrent high‐grade glioma requires a multidisciplinary approach. Re‐surgery^3^, ^4^ and re‐radiation^5^, ^6^, ^7^ offer some long‐term control but these options are feasible in few patients and salvage systemic therapy is required in the majority. Salvage systemic therapy currently revolves around the use of bevacizumab,^8^ lomustine (CCNU),^8^ and temozolomide.^9^ However, the results are far from satisfactory with these agents with median overall survival (OS) of 6.0‐8.0 months. Multiple molecules like lapatinib, sirolimus, temsirolimus, pazopanib, nintedanib, glufosfamide, imatinib, erlotinib, IFNβ, IFNβ with cis‐retinoic acid, menogaril, difluoro‐methyl ornithine, and sagopilone have been tried in studies but with dismal results.^10^

Mebendazole (Methyl 5‐benzoyl‐2‐benzimidazole carbamate) is an anti‐helminthic drug whose activity against GBM was discovered serendipitously.^11^ In vitro studies have confirmed the activity of mebendazole against GL261 mouse glioma line and 060919 human GBM line with the half‐maximal inhibitory concentration (IC50) of 0.3 and 0.1 µmol/L respectively.^12^ In vivo, it significantly extended mean survival (up to 63%) in syngeneic and xenograft orthotopic mouse glioma models.^12^ The mechanism of action of mebendazole is via reducing tubulin polymerization thus disrupting microtubule formation.^12^ The other speculated mechanisms of action are inhibition of vascular endothelial growth factor (VEGF), hypoxia‐inducible factor (HIF‐1alpha),^13^ and inactivation of B‐Cell lymphoma ‐2 (Bcl‐2).^14^, ^15^, ^16^ The molecular weight of mebendazole is 295 dalton (Da) and it crosses the blood‐brain barrier due to its lipophilic nature.^17^ In view of these features along with its promising activity in temozolomide resistant glioma cell lines.^1218^ We decided to repurpose mebendazole for the treatment of relapsed‐recurrent high‐grade gliomas. Hence, we conducted a phase 1 study with the primary objective to identify maximum tolerable dose (MTD) of mebendazole in combination with CCNU (CCNU+mebendazole), in combination with temozolomide (temozolomide+mebendazole) and in combination with re‐radiation and temozolomide (re‐radiation+temozolomide+mebendazole) respectively.

## 2 METHODS

### 2.1 Patients

Eligible patients had recurrent GBM, the diagnosis of recurrence was based on either histopathological confirmation or on unequivocal clinico‐radiological features. The confirmation of clinico‐radiological features was done in a neurooncology multidisciplinary board. There was no upper limit on the episode of recurrence, any recurrence postcompletion of primary treatment was included. The other eligibility criteria were age > 18 years, an Eastern Cooperative Oncology Group performance status (ECOG PS) score of 0, 1, 2, or 3 (on a scale from 0 to 5, with 0 indicating that the patient is fully active and higher scores indicating greater disability), normal organ and bone marrow functions ( Leukocytes ≥ 2000/mcL or absolute neutrophil count ≥ 1500/mcL, platelets ≥ 100 000/mcL, Total bilirubin < 1.5 × institutional upper limit of normal, serum glutamic‐oxaloacetic transaminase (SGOT)/serum glutamic‐pyruvic transaminase (SGPT)≤2.5 × institutional upper limit of normal and calculated creatinine clearance > 30 mL/min). Exclusion criteria included administration of any benzimidazole (albendazole, flubendazole, thiabendazole, fenbendazole, triclabendazole, etc) within the last 3 months, failure within 3 months of stopping temozolomide, presence of any uncontrolled comorbidities, presence of any medical or psychiatric condition which would have increased the risks associated with the study participation, interfered with investigational product administration, or interfered with the interpretation of the results. Pregnant ‐lactating females and patients with a history of previous life‐threatening complications with temozolomide were also excluded. The detailed inclusion and exclusion criteria are provided in the study protocol in the supplementary appendix.

### 2.2 Study design and oversight

The name of this study was Reverse swing‐M. This was a multiarm, open‐label, explanatory, phase 1 going to phase 2 study. Currently, phase 1 part has been completed and phase 2 is ongoing. The study protocol was approved by the local Institutional Ethics Committee (IEC) and was registered prospectively with Clinical Trials Registry‐ India (CTRI) {CTRI/2018/01/011542}. All patients provided written informed consent prior to participation. The study was conducted in accordance with the principles of the Declaration of Helsinki and the Good Clinical Practice guidelines of the International Conference on Harmonisation. Although the study was being conducted it was monitored by an independent data monitoring safety board.

The study was designed by VP and RJ. The data were analyzed and interpreted by VP & AB. VP wrote the first draft. All authors reviewed, revised, approved and took the decision to submit the manuscript for publication. All authors vouch for the accuracy, completeness of the data, and for the fidelity of the study to the study protocol. The study was funded by Brain Tumor Foundation (BTF) of India and the grant was awarded to VP. The funding agency had no role in study design, conduct, data collection or interpretation or writing of the paper.

### 2.3 Study interventions

**Figure 1:**
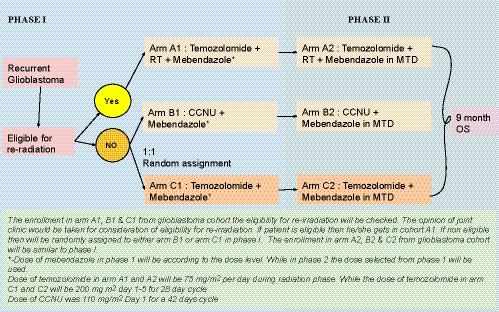
Depicts the study schema. MTD‐Maximum tolerable dose. OS‐Overall survival. CCNU‐ Lomustine.

All patients who were eligible for the study postscreening were discussed in the multidisciplinary neuro‐oncology tumor board. Patients were assessed for re‐irradiation in the meeting by the radiation oncologist. The eligibility criteria for re‐radiation were good performance status (ECOG PS < or =2) at re‐irradiation, prolonged time‐interval from the first course of irradiation (at least 2‐years), and recurrence confined to a single site in the supratentorial brain (moderate volume disease). Debulking surgery, though preferred, was not mandated at the time of recurrence/progression prior to re‐irradiation. If patients were ineligible for re‐irradiation then they were enrolled in either Arm B1 or Arm C1 of the study (Figure 1), While if they were eligible, they were enrolled in Arm A1 (Figure 1). The interventions in each of these arms are described below.

Arm A1‐ Salvage treatment entailed focal re‐irradiation of the recurrent disease/tumor‐bed with conservative margins using image‐guided intensity‐modulated radiation therapy (IMRT) on a 6 MV linear accelerator to a dose of 50.4‐54 Gy in 28‐30 fractions over 5.5‐6 weeks. Delineation of target volumes and organs‐at‐risk was done on appropriately acquired thin‐slice magnetic resonance imaging (MRI) sequences such as three‐dimensional fast spoiled gradient echo (FSPGR) and/or fluid attenuation inversion recovery (FLAIR) after co‐registration and fusion with axial planning computed tomography images that had been acquired in the supine position using customized thermoplastic immobilization of the skull. Oral temozolomide in a dose of 75 mg/m^2^ was administered concurrently throughout the course of re‐irradiation with ondansetron (8mg). The patients were assessed weekly during the treatment phase and thereafter 1 monthly till 1 year.

Arm B1‐ Lomustine (CCNU) was administered in a dose of 110 mg/m^2^ once on day 1 of a 42‐day cycle with ondansetron. The dose was rounded off to the nearest dose possible with a 40 mg capsule. Maximum of six cycles were administered. Patients were evaluated at 2 weekly intervals until six cycles were completed. Arm C1‐Temozolomide was administered in a dose of 200 mg/m^2^ once daily from day 1 to day 5, in a 28‐day cycle with ondansetron. Patients were evaluated at day 7, day 14, and day 28 of each cycle. A maximum of 12 cycles was administered.

Subsequent to treatment completion, the patients were followed up at 3 monthly intervals for the next 2 years, 6 monthly intervals for the 4‐5th year and yearly thereafter in each arm.

Mebendazole in each arm was administered as a 100 mg tablet. The dose in each arm was as per the dose level in which the patient was treated. The tablets were chewed with food by the patients. The dose modifications for CCNU, temozolomide, and mebendazole were done as per the study protocol section 9.

### 2.4 Mebendazole dose levels and escalation

**Figure 2:**
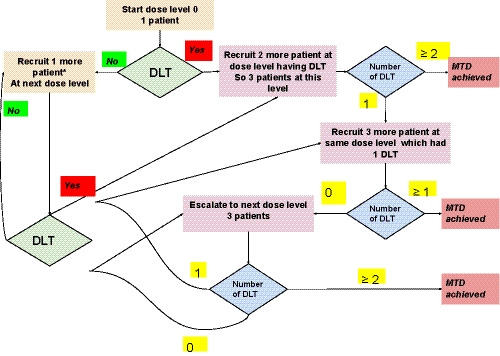
Depicts the accelerated titrated design 4 decision tree with the modified Fibonacci schema used in the current study. DLT‐Dose limiting toxicity and MTD‐Maximum tolerable dose. *Intrapatient dose escalation is allowed

The accelerated titrated design 4 provided by Simon and colleagues^19^ was used in the current study (Figures 1, 2 and 2). 100% dose escalation was allowed in this scheme in the titration phase.^20^ Escalating doses of oral mebendazole in the accelerated phase were as follows‐

1. Dose level 0:100 mg three times daily (TDS) per oral (PO)
2. Dose level 1:200 mg TDS PO
3. Dose level 2:400 mg TDS PO
4. Dose level 3:800 mg TDS PO
5. Dose level 4:1600 mg TDS PO

No further dose escalation was done over 1600 mg TDS as the number of tablets required (48 per day) made it unfeasible. In accordance with the accelerated titrated phase design 4, one patient was recruited at each dose level, with intrapatient dose escalation permitted, till a dose‐limiting toxicity (DLT) was seen (Figure 1).^19^ Once a DLT was seen, two more patients were recruited, making it three patients at that dose level as per the modified Fibonacci design. The decision for further dose escalation was taken in accordance with Figure 1. Further accelerations were made according to modified Fibonacci design escalation, ie 67%, 50%, 40%, and 30%–35% of the preceding doses.^20^ The escalation was rounded to the nearest dose which the strengths of the tablets allowed.

### 2.5 MTD, DLT and time

DLT was defined as grade 3‐4 life‐threatening adverse events related to the combination of the investigational drug and other chemotherapeutic agent(s) and/or radiation which occurred during the assigned period of the protocol. Adverse events were captured as per the Common Terminology Criteria for Adverse Events (CTCAE) version 4.03. The adverse events considered as DLT were neutropenia grade 4 or above, thrombocytopenia grade 4 or above, rise in SGPT or SGOT of grade 3 and persisting for 1 week and rise in SGPT or SGOT grade 4 or above. The occurrence of any one of the above was considered as DLT. DLT was captured over a period of one cycle in each arm which was over 1 week in arm A1, over 6 weeks in arm B1, and over 4 weeks in arm C1. Only the first cycle was considered for DLT assessments. DLT assessments were done weekly in Arm A1, 2 weekly (day 14, day 28, and day 42) in Arm B1 and in Arm C1 these assessments were done on day 7, day 14, and day 28. At each assessment, a complete hemogram, renal function test, and liver function tests were performed. In addition, at each visit, an inquiry was carried out for the presence of any clinical adverse events and compliance with the drugs was documented. MTD of mebendazole in each arm was the dose of mebendazole which was associated with two or more DLT in the respective arm.^20^

### 2.6 Statistical analysis

Descriptive statistics was performed. Continuous variables were expressed in terms of the median with range. Ordinal and nominal variables were expressed in terms of percentage with their corresponding 95% confidence interval (CI). The median progression‐free survival and overall survival were estimated using the Kaplan‐Meier method^21^ with the 95% confidence interval for the median constructed using the Brookmeyer and Crowley method.^22^ The progression‐free survival was defined as time in months from date of enrollment in the study to progression or death whichever was earlier. While the overall survival was defined as the time in months from date of enrollment in the study to death. The data cut‐off for the current analysis was 15th May 2019.

## 3 RESULTS

### 3.1 Baseline characteristics

The phase 1 recruited patients between 19th March 2018 and 11th January 2019. Eleven patients were enrolled in phase 1 of the 14 patients who were screened. Two patients were not willing to participate and one patient did not meet the eligibility criteria (Performance status 4) The number of patients required for dose‐finding in arm A1 was 1, arm B1 was 9 and in arm C1 was 1. The baseline characteristics are shown in Table 1. The baseline histopathology was glioblastoma in 6 (54.5), astrocytoma grade 2 or 3 in 2 (18.2) and oligodendroglioma grade 2‐3 in 3 (27.3) patients. The previous treatment details are shown in Table 1 and Table S1.

**Table 1:** Baseline characteristics

Variable	Value
Age‐years	
Median (Range)	46 (25‐68)
Gender‐no (%)	
Male	8 (72.7)
Female	3 (27.3)
ECOG PS‐no (%)	
0‐1	10 (90.9)
2‐3	1 (9.1)
NPS‐no (%)	
0‐1	10 (90.9)
2‐3	1 (9.1)
Comorbidities‐no (%)	
None^a^	10 (90.9)
Hypertension	1 (9.1)
Habits	
Smoker	1(9.1)
Non‐smoker	10(90.9)
Histopathology at baseline‐no (%)	
Glioblastoma IDH wild‐type	4 (36.3)
Glioblastoma IDH mutated	1 (9.1)
Glioblastoma NOS	1 (9.1)
Anaplastic Astrocytoma NOS	1 (9.1)
Diffuse Astrocytoma NOS	1 (9.1)
Anaplastic oligodendroglioma, IDH mutant and 1p/19q codeleted	2 (18.2)
Oligodendroglioma NOS	1 (9.1)
Previous surgery type‐no (%)	
Near Total	2 (18.2)
Subtotal	9 (81.8)
Previous radiation‐no (%)	
Yes	11(100%)
No	—
Previous radiation dose‐Gy	
Median (range)	59.4 (54‐64)
Previous chemotherapy‐no(%)	
No	—
Temozolomide	11 (100%)

Abbreviations: ECOG PS, Eastern Cooperative Oncology Group performance status; NPS, Neurological performance status.

^a^ Presence of type 2 diabetes mellitus, Obstructive pulmonary disease, Previous history of tuberculosis and presence of ischemic cardiac conditions were specifically sought, IDH ‐ Presence of Isocitrate dehydrogenase 1 and 2 mutations, NOS‐not otherwise specified.

### 3.2 Dose selection of mebendazole

In arm A1, no dose‐limiting toxicities were observed at any mebendazole dose level (Table 2). Therefore, the dose recommended for phase 2 was 1600 mg TDS of mebendazole with radiation and temozolomide. Similarly, in arm C1 too, no dose‐limiting toxicities were observed at any mebendazole dose level (Table 2). Therefore, the dose recommended for phase 2 was 1600 mg TDS of mebendazole with temozolomide.

**Table 2:** Table depicting the details of patients per dose level and the number of patients who reached MTD. N‐number of patients

		Dose level 0	Dose level 1	Dose level 2	Dose level 3	Dose level 4	Cumulative N
Arm A1	N	1^a^	1^a^	1^a^	1^a^	1^a^	1
	MTD reached	0	0	0	0	0	0
Arm B1	N	1^a^	1^a^	1^a^	1^a^ +5	1^a^ +2 + 1	9
	MTD reached	0	0	0	0	2	2
Arm C1	N	1^a^	1^a^	1^a^	1^a^	1^a^	1
	MTD reached	0	0	0	0	0	0

^a^ Patient undergoing intrapatient dose escalation, in each dose level the first patient reached upto dose level 4. MTD‐Maximum tolerable dose, Dose level 0‐ 100 mg TDS, Dose level 1‐ 200 mg TDS, Dose level 2‐ 400 mg TDS, Dose level 3‐ 800 mg TDS and Dose level 4‐ 1600 mg TDS of mebendazole.

In arm B1, no dose‐limiting toxicities were observed till the dose level 3 (800 mg TDS) (Table 2). However, during the intrapatient dose escalation, DLT was observed at dose level 4 (1600 mg TDS). The DLTs observed were grade 4 neutropenia and grade 3 thrombocytopenia. Hence in arm B1, the study entered modified Fibonacci design. Two more patients were entered at dose level 4 and no DLT was observed in these patients. As ⅓ patients had DLT, more patients had to be enrolled at this level. The 4th patient who was enrolled at this level had DLT. The DLTs observed were grade 3 neutropenia and grade 3 thrombocytopenia. As two of four patients had DLT at dose level 4 (1600 mg TDS), this dose level was considered as the MTD. As per the modified Fibonacci design, as only one patient was previously treated at 800mg TDS, an additional five patients were recruited (Study protocol‐Table 1). No DLT was observed in any of the patients at dose level 3. Hence the dose recommended for phase 2 was 800 mg TDS of mebendazole with CCNU.

### 3.3 Adverse events

Adverse events were captured in all patients. The details of adverse events for the whole study population of phase 1 during the DLT observation period and during the whole treatment period (cumulative rate of adverse events) are shown in Table 3. The adverse event in each arm, at each dose level during the DLT observation period are shown in Tables S2‐S5. Table 6 shows the interpatient variability in the occurrence of grade 1‐2 adverse events at different dose levels, the variance varied from 0‐0.911. Any grade adverse event and grade 3 or above adverse event were 100% (n = 11) and 18.2% (n = 2), respectively, during the DLT observation period. The incidence of any grade cumulative adverse events and grade 3 or above adverse events were 100% (n = 11) and 27.3% (n = 3) respectively during the whole study period. The three most common adverse events seen in the study were anemia (n = 9, 81.8%), nausea (n = 7, 63.6%), and fatigue (n = 6, 55.5%). The probability of the development of cumulative adverse events is shown in Figure S1.

**Table 3:** Details of adverse event during the dose limiting toxicity (DLT) observation period and during the whole study period (cumulative toxicity)

	Grade 1	Grade 2	Grade 3	Grade 4	Grade 5
Adverse events during the DLT observation phase					
Anemia	7 (63.6)	2 (18.2)	—	—	—
Neutropenia	1 (9.1)	1 (9.1)	1 (9.1)	1 (9.1)	—
Thrombocytopenia	1 (9.1)	—	2 (18.2)	—	—
FN	—	—	2 (18.2)	—	—
Rise in SGOT	1 (9.1)	—	—	—	—
Rise in SGPT	1 (9.1)	2 (18.2)	—	—	—
Rise in T bilirubin	2 (18.2)	—	—	—	—
Nausea	6 (54.5)	1 (9.1)	—	—	—
Vomiting	5 (45.5)	—	—	—	—
Mucositis	2 (18.2)	—	—	—	—
Diarrhea	—	2 (18.2)	—	—	—
Dyspepsia	1 (9.1)	—	—	—	—
Fatigue	3 (27.3)	1 (9.1)	—	—	—
Constipation	3 (27.3)	—	—	—	—
Insomnia	2 (18.2)	—	—	—	—
Anorexia	3 (27.3)	—	—	—	—
Adverse events during the whole treatment period					
Anemia	5 (45.5%)	2 (18.2)	2(18.2)	—	—
Neutropenia	1(9.1)	—	—	3(27.3)	—
Thrombocytopenia	1(9.1)	—	2(18.2)	1(9.1)	—
FN	—	—	3(27.3)	—	—
Rise in SGOT	1 (9.1)	—	—	—	—
Rise in SGPT	1 (9.1)	2 (18.2)	—	—	—
Rise in T bilirubin	2 (18.2)	—	—	—	—
Nausea	6 (54.5)	1 (9.1)	—	—	—
Vomiting	5 (45.5)	—	—	—	—
Mucositis	2 (18.2)	—	—	—	—
Diarrhea	—	2 (18.2)	1 (9.1)	—	—
Dyspepsia	1 (9.1)	1 (9.1)	—	—	—
Fatigue	5 (45.5)	1 (9.1)	—	—	—
Constipation	3 (27.3)	—	—	—	—
Insomnia	2 (18.2)	—	—	—	—
Anorexia	3 (27.3)	—	—	—	—
Alopecia	1 (9.1)	—	—	—	—
Maculopapular rash	1 (9.1)	—	—	—	—

Note

All adverse events are as per Common terminology criteria for adverse events (CTCAE) version 4.03. Maximum grade of adverse event is represented in the table.

### 3.4 Compliance, dose delay and dose reduction

Compliance with chemotherapy over the study period was 100% in all patients. Delay in the administration of chemotherapy was seen in four patients. The causes of delay were logistic issues in two patients and adverse events in two patients. The adverse events leading to delay were myelosuppression in both patients. Both these patients were in arm B1. These 2 patients required dose reductions in addition to dose delays. In both patients, two episodes of dose reduction were required and the maximum dose reduction done was 50% in one patient and 75% in another patient. The dose reduction was performed due to grade 3 and above myelosuppression in both patients.

The compliance with mebendazole was 100% in six patients (54.5%). Compliance in the other five patients was 33%, 86.6%, 97%, 97.6%, and 99.6% respectively. Dose delays in mebendazole were required in the same four patients in whom chemotherapy was delayed. Dose reduction for mebendazole was not required in the study.

### 3.5 Outcomes

**Figure 3:**
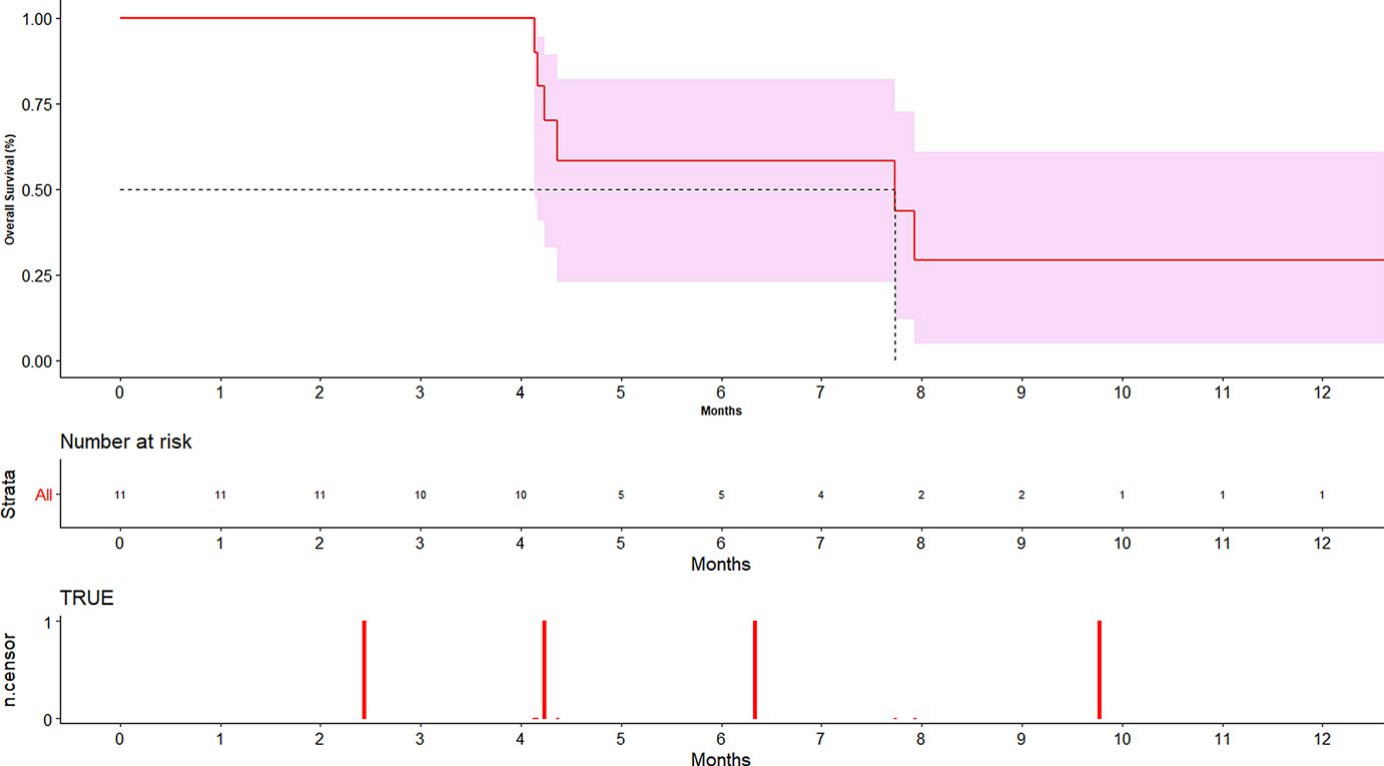
Depicting overall survival graph. X axis shows time in months. Y axis shows the percentage of patients. The shaded region depicts the 95% CI of overall survival curve

The median follow‐up was 9.77 months. The median PFS and OS were 6.33 (95% CI 2.43‐8.83) (Figure S2) and 7.73 (95% CI 4.13‐NA) months (Figure 3) respectively. The 9 month PFS and OS were 16.4% (95% CI 1.11‐48.3) and 29.2% (95% CI 4.7‐60.9) respectively.

## 4 DISCUSSION

This is probably the first systematic phase 1 study exploring the role of mebendazole in recurrent glioblastoma. The study provides the maximum tolerable dose of mebendazole in adults in combination with single‐agent temozolomide, single‐agent CCNU, and temozolomide‐radiation. The recommended phase 2 dose of mebendazole was 1600 mg TDS PO with single‐agent temozolomide and temozolomide radiation while it was 800 mg TDS PO with CCNU. These high doses attest to the favorable safety profile of this drug.

The reported IC50 in a human glioma cell line for mebendazole was 0.1 µmol/L and thus the objective was to achieve a brain concentration of mebendazole above this level.^12^ The brain to plasma ratio for mebendazole polymorphs B and C are 0.6 and 0.8 respectively.^17^ Thus, a plasma level of > 0.17 µmol/L was necessary for the achievement of adequate levels in the brain. Chronic administration of 1.5 g per day and 40 mg/kg per day (2.4 g per day for 60 kg) of mebendazole is known to produce a mean peak plasma level of 0.28 μmol/L ( ±0.14 μmol/L) and 0.47 µmol/L (0.34‐1.69 μmol/L) respectively.^23^, ^24^ Thus, we hypothesized that dose level 2 (400 mg TDS = 1.2 g per day) and above should produce adequate levels of mebendazole for its cytotoxic activity. However, still, we selected higher doses for titration as interpatient and intrapatient variation^25^ is high in mebendazole and a higher dose would enable us to achieve IC50 concentrations in most of the patients. The dose of 1600 mg TDS (4.8 g per day) was the highest dose beyond which no titration was performed as the available strength of mebendazole was 100 mg and it would have been impractical to dose further with this strength.^8^, ^26^, ^27^, ^28^.

The rate of adverse events was low in the current study. No DLT was observed with the addition of mebendazole to temozolomide and temozolomide‐radiation combination. While the DLTs observed with CCNU were neutropenia and thrombocytopenia, whether the DLT was because of CCNU or mebendazole is an open question. CCNU in a dose of 110 mg/m^2^ is toxic and in the authors’ previous experience it alone can lead to any grade myelosuppression in 80% and grade 3‐4 myelosuppression in 33% of patients.^29^ Myelosuppression and liver dysfunction are both speculated side effects of high dose mebendazole.^18^ Hence, these acute adverse events were included in the definition of DLT. However, in the current study, there was no episode of grade 3 or above liver dysfunction in any arm.

Late adverse events, especially in arm A1, were not considered for inclusion in the definition of DLT. This decision was based on multiple reasons. This was done purposefully as these late events start occurring 3 months postradiation completion and as the median PFS with re‐radiation in glioma is below 6 months.^30^, ^31^ The investigators felt that decision based on late‐onset events would be not clinically relevant as most patients would progress before their occurrence. Further its assessment would have been complicated by the adverse events of adjuvant temozolomide, which these patients would receive. Considering late adverse events would have also denied the opportunity for intrapatient dose escalation. Lastly there was no conclusive data (in vivo or in vitro) to suggest that the addition of mebendazole to radiation leads to radiosensitization. Hence, we selected traditional acute adverse events even in arm A1 and the duration was limited to 1 week. The duration of 1 week was selected as it is customary in solid tumor patients undergoing chemoradiation to be assessed weekly at our centre^32^ and dose adjustments of concurrent chemotherapy are made based on adverse events seen in last week.

Currently to the best of authors’ knowledge three more phase 1 studies are running across the globe, repurposing mebendazole in the treatment of gliomas. Two of these phase 1 studies (NCT02644291, NCT01837862) are in the pediatric population, one dealing with recurrent‐progressive pediatric brain tumors and the latter in recurrent‐progressive gliomas. One is exploring the use of mebendazole in newly diagnosed high‐grade glioma patients treated with temozolomide (NCT01729260). The results of these studies are expected in 2020‐2021. As opposed to these studies, the current study used a more practical approach and repurposed mebendazole in a holistic way with permutations and combinations commonly used in recurrent gliomas. In addition, the current phase 1 used the accelerated titrated design which is considered as a more efficient design than the traditional 3+3 approach. This design allows for intrapatient dose escalation, allows rapid dose escalation, exposes a lower proportion patients to very low doses of a drug and is time efficient.^19^

Pharmacokinetic analysis was not performed in the current study. Mebendazole is an old drug with well‐established pharmacokinetic parameters. The recommended dose of mebendazole varies from 100 mg single tablet in pinworm to 40‐50 mg/kg per day for 6‐24 months in echinococcosis.^11^ Hence, investigators felt that doing a pharmacokinetic study during phase 1 is unlikely to add any new information.

The administration of mebendazole was oral and while it is a favorable aspect it does limit the applicability of this drug in patients who cannot tolerate enteral feeding or require a nasogastric tube. Mebendazole dose was administered in the current study in 100 mg tablets. Hence ensuring compliance for the administration of 800‐1600 mg TDS can be an issue and requires the patient to be highly motivated. In the current study, we had a few patients who initially had ODG histology with 1p/19q deletion (n = 2), and while this may have skewed the PFS/OS, it does not impact the primary objective of phase 1 as we are looking at toxicity.^33^, ^34^

## 5 CONCLUSION

Mebendazole can be combined with single‐agent temozolomide, single‐agent CCNU, and a combination of temozolomide with radiation. The recommended phase 2 dose of mebendazole is 1600 mg TDS with temozolomide and temozolomide‐radiation combination while a dose of 800 mg TDS should be used with single‐agent CCNU.

## CONFLICT OF INTEREST

None.

## Supporting information

Appendix S1

## Data Availability

Data will be made available for any one requesting the data with an ethics approved project for scientific use in accordance with the countries data sharing norms.
