# Supplementary material for: Reverse swing‐M, phase 1 study of repurposing mebendazole in recurrent high‐grade glioma
Source: Cancer Med. 2020 May 13;9(13):4676–85. doi: 10.1002/cam4.3094 (PMC7333848; doi:10.1002/cam4.3094)
Supplement: Supplementary file 1 — Appendix S1 [file CAM4-9-4676-s001.pdf]

Table of Contents

Supplementary table 1 2

Supplementary table 2- Arm A1 4

Supplementary table 3- Arm B1 8

Supplementary table 4- Arm C1 12

Supplementary table 5 16

Supplementary table 6 17

Supplementary figure 1 18

Supplementary figure 2 19

Supplementary table 1

Patient Number	Previous treatment details
1	Primary treatment-Subtotal resection followed by adjuvant radiation with a dose of 59.4 Gy (1.8 Gy per fraction) with concurrent temozolomide (75 mg/m2 daily) followed by adjuvant temozolomide.<br>Enrolled at first recurrence
2	Primary treatment-Subtotal resection followed by adjuvant radiation with a dose of 59.4 Gy (1.8 Gy per fraction) concurrent temozolomide (75 mg/m2 daily) followed by adjuvant temozolomide .<br>Enrolled at first recurrence
3	Primary treatment-Subtotal resection followed by adjuvant radiation with a dose of 60 Gy (2 Gy per fraction) over 30 # with concurrent temozolomide (75 mg/m2 daily) followed by adjuvant temozolomide.<br>Enrolled at first recurrence
4	Primary treatment-Near total resection followed by adjuvant radiation with a dose of 54 Gy (1.8 Gy per fraction).<br>First recurrence- Surgery followed by adjuvant temozolomide<br>Second recurrence- Surgery followed by reradiation<br>Enrolled at third recurrence
5	Primary treatment-Near total resection followed by adjuvant radiation with a dose of 59.4 Gy (1.8 Gy per fraction) with concurrent temozolomide (75 mg/m2 daily) followed by adjuvant temozolomide .<br>Enrolled at first recurrence
6	Primary treatment- Subtotal total resection followed by adjuvant radiation with a dose of 55.8 Gy (1.8 Gy per fraction) with concurrent temozolomide (75 mg/m2 daily) followed by adjuvant temozolomide .<br>Enrolled at first recurrence
7	Primary treatment- Subtotal total resection followed by adjuvant radiation with a dose of 55.8 Gy (1.8 Gy per fraction) with concurrent temozolomide (75 mg/m2 daily) followed by adjuvant temozolomide .<br>Enrolled at first recurrence
8	Primary treatment-Subtotal resection followed by adjuvant radiation with a dose of 59.4 Gy (1.8 Gy per fraction) with concurrent temozolomide (75 mg/m2 daily) followed by adjuvant temozolomide .<br>Enrolled at first recurrence

9	Primary treatment-Subtotal resection followed by adjuvant radiation with a dose of 59.4 Gy (1.8 Gy per fraction) with concurrent temozolomide (75 mg/m2 daily) followed by adjuvant temozolomide .<br>Enrolled at first recurrence
10	Primary treatment- Subtotal resection followed by adjuvant radiation with a dose of 64 Gy (2 Gy per fraction) with concurrent temozolomide (75 mg/m2 daily)<br>Enrolled at first recurrence
11	Primary treatment-Subtotal resection followed by adjuvant radiation with a dose of 59.4 Gy (1.8 Gy per fraction) with concurrent temozolomide (75 mg/m2 daily) followed by adjuvant temozolomide<br>First recurrence- Resurgery<br>Enrolled at second recurrence

Supplementary table 1- Table depicts the treatment received prior to the enrollment in the study.
Gy-Gray

Supplementary table 2- Arm A1

Dose level - 0 (n=1)					
	Grade 1	Grade 2	Grade 3	Grade 4	Grade 5
Anemia	-	-	-	-	-
Neutropenia	-	-	-	-	-
Thrombocytopenia	-	-	-	-	-
FN	-	-	-	-	-
Rise in creatinine	-	-	-	-	-
Rise in SGOT	-	-	-	-	-
Rise in SGPT	-	-	-	-	-
Rise in T bilirubin	-	-	-	-	-
Nausea	-	-	-	-	-
Vomiting	-	-	-	-	-
Mucositis	-	-	-	-	-
Diarrhea	-	-	-	-	-
Dyspepsia	-	-	-	-	-
Constipation	1	-	-	-	-
Dose level 1(n=1)					
Anemia	-	-	-	-	-
Neutropenia	-	-	-	-	-
Thrombocytopenia	-	-	-	-	-
FN	-	-	-	-	-
Rise in creatinine	-	-	-	-	-
Rise in SGOT	-	-	-	-	-

Rise in SGPT	-	-	-	-	-
Rise in T bilirubin	-	-	-	-	-
Nausea	-	-	-	-	-
Vomiting	-	-	-	-	-
Mucositis	-	-	-	-	-
Diarrhea	-	-	-	-	-
Dyspepsia	-	-	-	-	-
Dose level 2 (n=1)					
Anemia	-	-	-	-	-
Neutropenia	-	-	-	-	-
Thrombocytopenia	-	-	-	-	-
FN	-	-	-	-	-
Rise in creatinine	-	-	-	-	-
Rise in SGOT	-	-	-	-	-
Rise in SGPT	-	-	-	-	-
Rise in T bilirubin	-	-	-	-	-
Nausea	-	-	-	-	-
Vomiting	-	-	-	-	-
Mucositis	-	-	-	-	-
Diarrhea	-	-	-	-	-
Dyspepsia	-	-	-	-	-
Fatigue	1	-	-	-	-
Dose level 3 (n=1)					
Anemia	-	-	-	-	-
Neutropenia	-	-	-	-	-
Thrombocytopenia	-	-	-	-	-

FN	-	-	-	-	-
Rise in creatinine	-	-	-	-	-
Rise in SGOT	-	-	-	-	-
Rise in SGPT	-	-	-	-	-
Rise in T bilirubin	-	-	-	-	-
Nausea	-	-	-	-	-
Vomiting	-	-	-	-	-
Mucositis	-	-	-	-	-
Diarrhea	-	-	-	-	-
Dyspepsia	-	-	-	-	-
Fatigue	1	-	-	-	-
Dose level 4 (n=1)					
Anemia	-	-	-	-	-
Neutropenia	-	-	-	-	-
Thrombocytopenia	-	-	-	-	-
FN	-	-	-	-	-
Rise in creatinine	-	-	-	-	-
Rise in SGOT	-	-	-	-	-
Rise in SGPT	-	-	-	-	-
Rise in T bilirubin	-	-	-	-	-
Nausea	-	-	-	-	-
Vomiting	-	-	-	-	-
Mucositis	-	-	-	-	-
Diarrhea	-	-	-	-	-
Dyspepsia	-	-	-	-	-
Fatigue	1	-	-	-	-

Constipation	1	-	-	-	-

Supplementary table -2: Details of adverse event during the dose limiting toxicity (DLT) observation period in arm A1. All adverse events are as per common terminology criteria for adverse events (CTCAE) version 4.03. Maximum grade of adverse event is represented in the table.

Supplementary table 3- Arm B1

Dose level 0 (n=1)					
	Grade 1	Grade 2	Grade 3	Grade 4	Grade 5
Anemia	-	-	-	-	-
Neutropenia	-	-	-	-	-
Thrombocytopenia	-	-	-	-	-
FN	-	-	-	-	-
Rise in creatinine	-	-	-	-	-
Rise in SGOT	-	-	-	-	-
Rise in SGPT	-	-	-	-	-
Rise in T bilirubin	-	-	-	-	-
Nausea	1	-	-	-	-
Vomiting	-	-	-	-	-
Mucositis	1	-	-	-	-
Diarrhea	-	-	-	-	-
Dyspepsia	1	-	-	-	-
Constipation	-	-	-	-	-
Dose level 1(n=1)					
Anemia	-	-	-	-	-
Neutropenia	-	-	-	-	-
Thrombocytopenia	-	-	-	-	-
FN	-	-	-	-	-
Rise in creatinine	-	-	-	-	-
Rise in SGOT	-	-	-	-	-
Rise in SGPT	-	-	-	-	-

Rise in T bilirubin	-	-	-	-	-
Nausea	-	-	-	-	-
Vomiting	-	-	-	-	-
Mucositis	-	-	-	-	-
Diarrhea	-	-	-	-	-
Dyspepsia	1	-	-	-	-
Constipation	1	-	-	-	-
Fatigue	1	-	-	-	-
Anorexia	1	-	-	-	-
Dose level 2 (n=1)					
Anemia	-	-	-	-	-
Neutropenia	-	-	-	-	-
Thrombocytopenia	-	-	-	-	-
FN	-	-	-	-	-
Rise in creatinine	-	-	-	-	-
Rise in SGOT	-	-	-	-	-
Rise in SGPT	-	-	-	-	-
Rise in T bilirubin	-	-	-	-	-
Nausea	1	-	-	-	-
Vomiting	-	-	-	-	-
Mucositis	-	-	-	-	-
Diarrhea	1	-	-	-	-
Dyspepsia	1	-	-	-	-
Fatigue	1	-	-	-	-
Fever	1	-	-	-	-
Dose level 3(n=6*)					

Anemia	3	-	-	-	-
Neutropenia	-	-	-	-	-
Thrombocytopenia	1	-	-	-	-
FN	-	-	-	-	-
Rise in creatinine	-	-	-	-	-
Rise in SGOT	1	-	-	-	-
Rise in SGPT	1	1	-	-	-
Rise in T bilirubin	-	-	-	-	-
Nausea	2	1	-	-	-
Vomiting	3	-	-	-	-
Mucositis	-	-	-	-	-
Diarrhea	-	-	-	-	-
Dyspepsia	1	-	-	-	-
Fatigue	1	-	-	-	-
Anorexia	1				
Dose level-4 (n=4*)					
Anemia	3	2	-	-	-
Neutropenia	1	1	1	1	-
Thrombocytopenia	1	-	2	-	-
FN	-	-	2	-	-
Rise in creatinine	-	-	-	-	-
Rise in SGOT	-	-	-	-	-
Rise in SGPT	-	2	-	-	-
Rise in T bilirubin	2	-	-	-	-
Nausea	2	-	-	-	-
Vomiting	1	-	-	-	-

Mucositis	2	-	-	-	-
Diarrhea	-	2	-	-	-
Dyspepsia	1	-	-	-	-
Fatigue	1	1	-	-	-
Insomnia	1	-	-	-	-

Supplementary table -3: Details of adverse event during the dose limiting toxicity (DLT) observation period in arm B1. All adverse events are as per common terminology criteria for adverse events (CTCAE) version 4.03. Maximum grade of adverse event is represented in the table.

Supplementary table 4- Arm C1

Dose level 0(n=1)					
	Grade 1	Grade 2	Grade 3	Grade 4	Grade 5
Anemia	1	-	-	-	-
Neutropenia	-	-	-	-	-
Thrombocytopenia	-	-	-	-	-
FN	-	-	-	-	-
Rise in creatinine	-	-	-	-	-
Rise in SGOT	-	-	-	-	-
Rise in SGPT	-	-	-	-	-
Rise in T bilirubin	-	-	-	-	-
Nausea	-	-	-	-	-
Vomiting	-	-	-	-	-
Mucositis	-	-	-	-	-
Diarrhea	-	-	-	-	-
Dyspepsia	-	-	-	-	-
Constipation	1	-	-	-	-
Dose level 1(n=1)					
Anemia	-	-	-	-	-
Neutropenia	-	-	-	-	-
Thrombocytopenia	-	-	-	-	-
FN	-	-	-	-	-
Rise in creatinine	-	-	-	-	-
Rise in SGOT	-	-	-	-	-
Rise in SGPT	-	-	-	-	-

Rise in T bilirubin	-	-	-	-	-
Nausea	1	-	-	-	-
Vomiting	1	-	-	-	-
Mucositis	-	-	-	-	-
Diarrhea	-	-	-	-	-
Dyspepsia	-	-	-	-	-
Dose level 2(n=1)					
Anemia	-	-	-	-	-
Neutropenia	-	-	-	-	-
Thrombocytopenia	-	-	-	-	-
FN	-	-	-	-	-
Rise in creatinine	-	-	-	-	-
Rise in SGOT	-	-	-	-	-
Rise in SGPT	-	-	-	-	-
Rise in T bilirubin	-	-	-	-	-
Nausea	1	-	-	-	-
Vomiting	-	-	-	-	-
Mucositis	-	-	-	-	-
Diarrhea	-	-	-	-	-
Dyspepsia	-	-	-	-	-
Fatigue	1	-	-	-	-
Dose level 3(n=1)					
Anemia	-	-	-	-	-
Neutropenia	-	-	-	-	-
Thrombocytopenia	-	-	-	-	-
FN	-	-	-	-	-

Rise in creatinine	-	-	-	-	-
Rise in SGOT	-	-	-	-	-
Rise in SGPT	-	-	-	-	-
Rise in T bilirubin	-	-	-	-	-
Nausea	-	-	-	-	-
Vomiting	-	-	-	-	-
Mucositis	-	-	-	-	-
Diarrhea	-	-	-	-	-
Dyspepsia	-	-	-	-	-
Fatigue	1	-	-	-	-
Insomnia	1	-	-	-	-
Dose level 4(n=1)					
Anemia	1	-	-	-	-
Neutropenia	-	-	-	-	-
Thrombocytopenia	-	-	-	-	-
FN	-	-	-	-	-
Rise in creatinine	-	-	-	-	-
Rise in SGOT	-	-	-	-	-
Rise in SGPT	-	-	-	-	-
Rise in T bilirubin	-	-	-	-	-
Nausea	1	-	-	-	-
Vomiting	-	-	-	-	-
Mucositis	-	-	-	-	-
Diarrhea	-	-	-	-	-
Dyspepsia	-	-	-	-	-
Fatigue	1	-	-	-	-

Anorexia	1	-	-	-	-
Alopecia	1	-	-	-	-

Supplementary table -4: Details of adverse event during the dose limiting toxicity (DLT) observation period in arm C1. All adverse events are as per common terminology criteria for adverse events (CTCAE) version 4.03. Maximum grade of adverse event is represented in the table.

Supplementary table 5

	Grade 1	Grade 2	Grade 3	Grade 4	Grade 5
Anemia	4	2	-	-	-
Neutropenia	1	1	1	1	-
Thrombocytopenia	1	-	2	-	-
FN	-	-	2	-	-
Rise in creatinine	-	-	-	-	-
Rise in SGOT	-	-	-	-	-
Rise in SGPT	-	2	-	-	-
Rise in T bilirubin	2	-	-	-	-
Nausea	3	-	-	-	-
Vomiting	1	-	-	-	-
Mucositis	2	-	-	-	-
Diarrhea	-	2	-	-	-
Dyspepsia	1	-	-	-	-
Fatigue	3	1	-	-	-
Insomnia	1	-	-	-	-
Anorexia	1	-	-	-	-
Alopecia	1	-	-	-	-
Constipation	1	-	-	-	-

Supplementary table -5: Details of adverse event during the dose limiting toxicity (DLT) observation period in the dose level 4 (highest dose). N=6. All adverse events are as per common terminology criteria for adverse events (CTCAE) version 4.03. Maximum grade of adverse event is represented in the table.

Supplementary table 6

**Dose level**	**100**	**200**	**400**	**800**	**1600**
Variance	0	0.653	0.653	0.911	0

Supplementary table 6- Interpatient variability measurement on different dose levels.

Supplementary figure 1

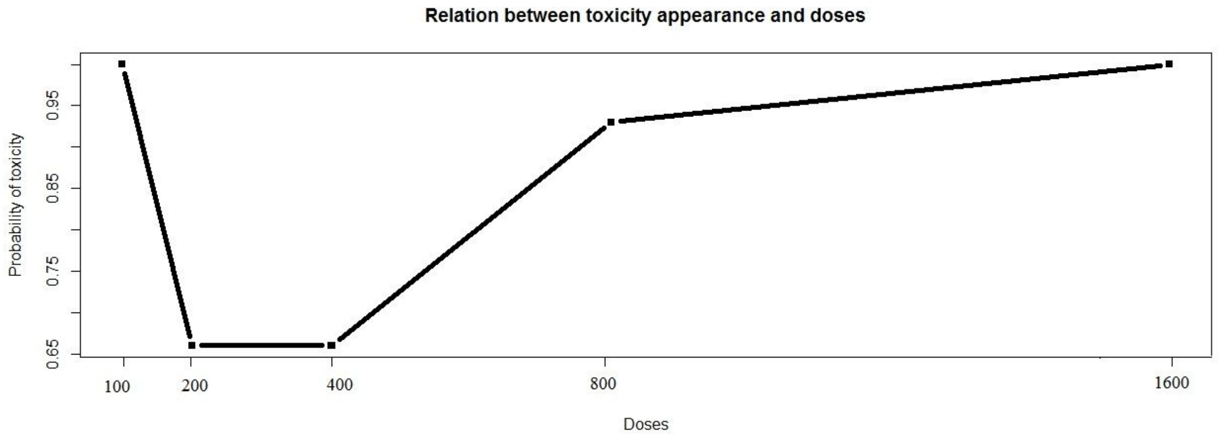

Supplementary figure 1-Probability of development of grade 1-2 adverse events at each dose level.

Supplementary figure 2

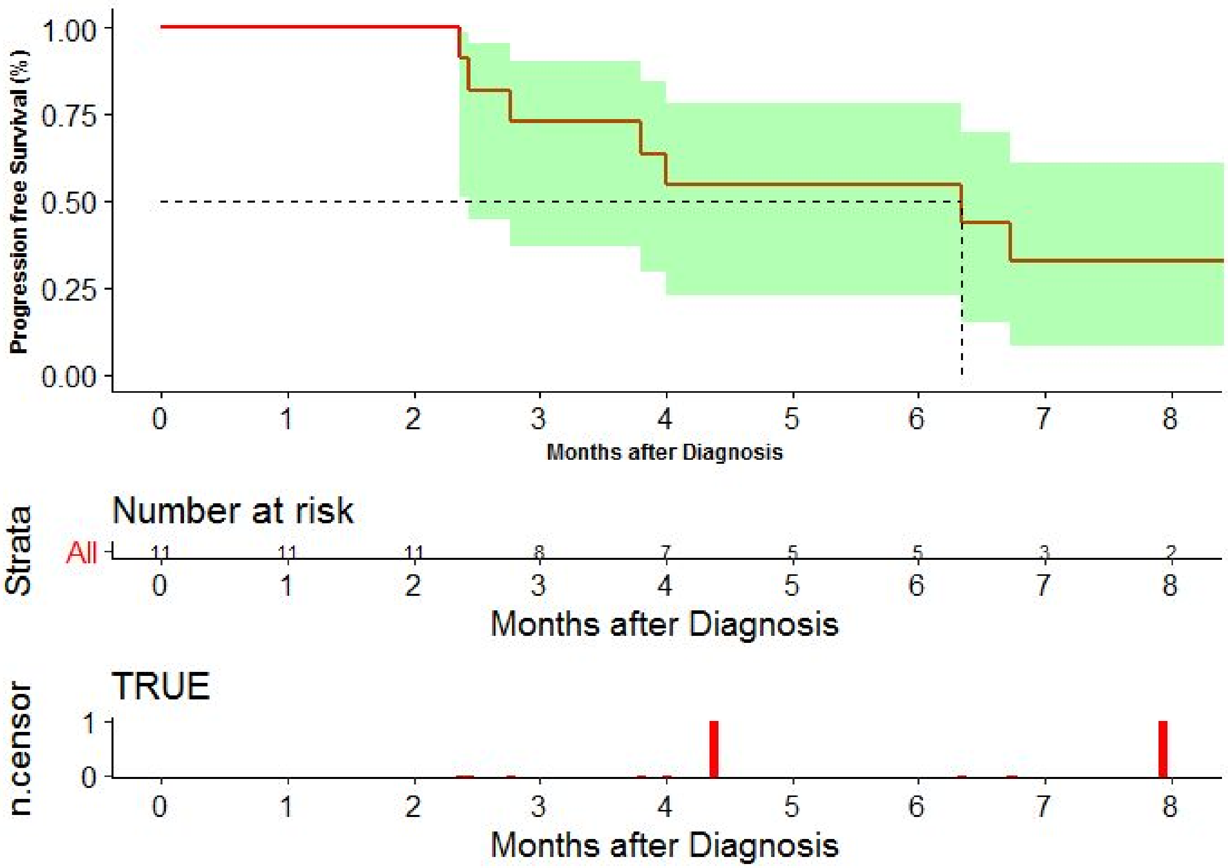

Supplementary figure 2-Progression free survival graph. X axis shows time in months. Y axis shows the percentage of patients. The shaded region depicts the 95%CI of progression free survival curve.
